# Preliminary Investigation of Essentially Derived Variety of Tea Tree and Development of SNP Markers

**DOI:** 10.3390/plants12081643

**Published:** 2023-04-13

**Authors:** Li Li, Xiangru Li, Fei Liu, Jialin Zhao, Yan Zhang, Weiming Zheng, Li Fan

**Affiliations:** 1 College of Tea and Food Science, Wuyi University, 358# Baihua Road, Wuyishan 354300, China; lixr@fafu.edu.cn (X.L.); 18815906898@163.com (F.L.); zjl123450913@163.com (J.Z.); 18296018498@163.com (Y.Z.); tseng0524@163.com (W.Z.); 2 College of Horticulture, Fujian Agriculture and Forestry University, 15# Shangxiadian Road, Fuzhou 350002, China

**Keywords:** essentially derived variety, genotyping by sequencing, genetic similarity, identification markers

## Abstract

The continuous emergence of Essentially Derived Varieties (EDVs) in the process of tea tree breeding will endanger and affect the innovation ability and development potential of tea tree breeding. In this study, genotyping by sequencing (GBS) technology was used to screen high-quality genomic SNPs for the first time to investigate the derived relationships of 349 tea trees from 12 provinces in China. A total of 973 SNPs uniformly covering 15 tea tree chromosomes with high discrimination capacity were screened as the core SNP set. A genetic similarity analysis showed that 136 pairs of tea trees had a genetic similarity coefficient (GS) > 90%, among which 60 varieties/strains were identified as EDVs, including 22 registered varieties (19 were indisputably EDVs). Furthermore, 21 SNPs with 100% identification of 349 tea trees were selected as rapid identification markers, of which 14 SNP markers could be used for 100% identification of non-EDV. These results provide the basis for the analysis of the genetic background of tea trees in molecular-assisted breeding.

## 1. Introduction

True breeding innovation is a process that requires a large amount of investment and careful long-term research. However, some formal “new varieties” are constantly being bred around a few limited breeding resources. These “new varieties” have a single genetic background, resulting in the loss of population genetic diversity. The main reason is that these “new varieties” are all inbred offspring of a similar initial variety (IV), and they retain the essential characteristics from the genotype or combination of genotypes of the IV. These “new varieties” are called Essentially Derived Varieties (EDVs) [1]. At present, ornamental plants, fruit trees, rice, and wheat have been reported to have a lot of “new varieties”, mainly through mutation and genetic modification; the genetic background of these EDVs has a serious homogeneity phenomenon [2,3,4,5]. The registration of these EDVs as a new variety will damage the interests of the IV owners, reduce the enthusiasm of the original breeding innovators, and cause the proliferation of cosmetic breeding, which is not conducive to the real improvement of agricultural production characteristics. In addition, due to the high genetic similarities between EDVs and IV, the existence of a large number of EDVs narrows the genetic basis, which is also not conducive to further genetic improvement [6]. As a result of the proliferation of EDVs, true new varieties will inevitably become increasingly scarce and breeding innovation will decrease, resulting in a vicious circle. Therefore, the identification and control of derivative varieties is an important problem to be solved.

With the development of domestic and international seed trade, the commercial quality of seeds based on authenticity and purity is becoming more and more important for both breeders and farmers [7]. Since the concept of EDV was introduced into the UPOV Act in 1991, there have been studies on the assessment and identification of EDVs for some crops [3]. China was the first country to grow tea trees [8], but systematic research on tea trees in this aspect is relatively blank. China officially implemented the EDV system in March 2022, so it is necessary to investigate the current situation of tea plant EDVs [9,10].

Due to the nature of self-incompatibility, the intra-specific hybridization of tea trees is very frequent [11]. Therefore, the occurrence of mutants in tea varieties is a common phenomenon. The standard morphological and physiological characters used to register or grant plant breeders’ rights are adequate to determine the distinctness, uniformity and stability (DUS) of the new mutant variety. However, these characteristics do not seem well suited to associating mutants with IV, as they do not seem to allow accurate determination of genetic conformity [12]. As a result, a mutant different from the IV phenotype is likely to be identified as a new variety, whereas previous studies have shown that morphologically different tea trees are likely to be genetically similar in nature [13].

DNA detection technology is an indispensable technical guarantee for effectively implementing the EDV system. In previous studies, molecular markers, such as amplified fragment length polymorphisms (AFLPs) and simple sequence repeats (SSRs), had been used to provide a method of determining genetic similarity (GS) and thus distinguishing EDVs [14,15,16]. With the development of biotechnology, Single Nucleotide Polymorphisms (SNPs) are considered the preferred markers for EDV evaluation due to their ability to detect more polymorphisms and their simplicity, speed and suitability for automation [17,18,19,20]. The screening of SNP sites is key to the development of SNP molecular markers. GBS technology is a rapid, flexible, low-cost, and robust high-throughput genotyping method for screening SNP molecular markers and genotyping, which can simultaneously discover a large number of SNP variants and call genotypes within a broad range of samples with high accuracy. These characteristics make GBS an excellent genotyping and SNP discovery technique for studying genetic diversity and the molecular breeding of crops [21].

Compared to major food crops, SNP molecular markers in tea trees developed late and need to be further improved and supplemented. The development of tea plant SNP molecular markers and the establishment of real and reliable DNA molecular detection technology are of great strategic significance for the implementation of the EDV system. In this study, 349 tea trees from 12 provinces in China were collected for whole-genome GBS high-throughput sequencing, and high-quality SNPs were used to analyze the genetic similarity of 349 trees. Meanwhile, a set of simple and rapid molecular markers for SNP identification of tea trees was developed. This is the first large-scale investigation on the EDV of tea trees, which provides a reference for the effective implementation of the EDV system.

## 2. Materials and Methods

### 2.1. Sample and DNA Isolation

Samples were collected from the Tea Germplasm Resource Park of Wuyi University (N 27°61′01.34″, E 117°96′63.51″) and the Tea Germplasm Resources Reserve of the Fujian Province (N 27°43′42.46″, E 118°0′14.40″); a total of 349 tea trees from 12 provinces in China, including 63 national elite cultivars, 58 provincial elite cultivars and 228 landraces, were used in the study. Six pairs of tea trees from the same variety were used as positive controls for the essentially derived relationship. All samples used are shown in Table S1. Relevant information on tea plant breeding was taken from the book *Tea Plant Varieties in China* [22] and *Tea Plant Clonal Varieties in China* [23].

DNA was extracted using the plant genomic DNA extraction kit (KangWei CW0553S, China), and the concentration and purity of the DNA were determined by NanoDrop ultraviolet spectrophotometer (Thermo Scientific, Carlsbad, CA, USA). DNA integrity was detected by 0.8% agarose electrophoresis. The extracted genomic DNA was stored at −80 ℃.

### 2.2. Genotyping by Sequencing (GBS)

GBS library construction was done according to Wu [24]. Briefly, the genomic DNA was digested using restriction enzymes (*EcoR* I and *Nia* III) and then ligated with barcoded adapters and common Illumina sequencing adaptors. A total of 349 multiplex libraries corresponding to 349 tea trees were constructed, with each library DNA sample having a unique nucleotide multiplex identifier for the barcode adapter. These libraries were subjected to Pair-end sequencing using the Illumina NovaSeq 6000 sequencing platform (150 bp*2, Shanghai Biozeron Co., Ltd.).

Trimmomatic with Minlen 75 (version: 0.36, Jülich, Germary) was used to trim and quality control the raw paired-end reads [25]. BWA-MEM (version 0.7.10) [26] was used to align high-quality sequencing reads to the tea plant reference genome sequence [27]. SamTools (version 1.9) was used to convert sam files to bam files [28]. GATK (4.1.2.0) was used to call SNPs [29]. The initial SNP quality filtering criteria were QD ≥ 2.0, FS ≤ 60.0, MQ ≥ 40.0, Mapping Quality Rank Sum ≥ −12.5 and Read Pos Rank Sum≥ −8.0. VCF tools (version 0.1.11) were used to further filter the SNPs with the criteria: ‘- minQ 20 -minDP 4′ [30]. SNPs with minor allele frequency (MAF) > 0.1, missing data <20% and no sequence variation in the flanking region of 150 bp were selected as candidate SNPs. Finally, a total of 12,937,679 SNPs were retained in 349 tea trees (Tables S1 and S2).

When examining genetic similarities between moderately or closely related germplasm, the required number of SNPs may be more than 300, with relatively high PIC values and uniform genomic coverage [18]. Therefore, an optimal core SNP set with MAF > 0.15, miss rate ≤ 5%, PIC > 0.15, more than 300 SNPs and uniform coverage of the whole genome was selected for further analysis. The observed heterozygosity (Ho), minor allele frequency (MAF) and Polymorphic information content (PIC) were calculated using PowerMaker 3.25 software (Raleigh, NC, USA) [31].

### 2.3. Population Structure Analysis

Population structure was analyzed by Admixture software [32]. All tea trees were assigned into corresponding groups based on the values of the membership coefficient. The optimal K value was determined according to cross-validation error (CV error). Principal component analysis (PCA) was performed using Plink software, with the principal components plotted against one another [33] using R 3.4 to visualize patterns of genetic variation. A phylogenetic NJ-tree was constructed by the MEGA software, and the color of the phylogenetic tree was coded by iTOL [34].

### 2.4. Genetic Similarity Analysis

A genetic similarity analysis was carried out for pairs of samples using the NTSYSpc2.11 software [35] to calculate the distribution frequency of SNPs and the genetic similarity coefficient (GS), GS = NS/(NS + ND): NS is the same number of SNP genotypes, and ND is the different SNP genotypes [36].

According to the recommendations of the International Seed Federation (ISF) [37], a GS value greater than 0.9 would represent strong evidence of essential derivation. Therefore, the pairs of samples with GS > 0.9 are considered likely to have an essentially derived relationship. Six pairs of samples of the same variety from different growing places were used as positive controls. When the GS value of any pair of samples was greater than that of the positive controls, this pair of samples is considered to have an indisputable essentially derived relationship. Varieties with late registration dates are considered EDV.

### 2.5. Core Varieties/Strains Analysis

According to Wang et al., 2007 [38], the least distance stepwise sampling method was used to construct the core germplasm, which could preserve the genetic diversity of the original population to the maximum extent. A pairwise comparison matrix by calculating the numbers of differential SNP genotypes between each collection was built within each population; the missing genotype was treated as null. Fewer differential SNP genotypes indicated closer kinship with others. The top 20% of collections with close kinship were considered core varieties in each population.

Cluster is one of the important factors that will affect the results of core collection [38]; collections with extremely admixture backgrounds were excluded from this analysis based on the phylogenetic and the population structure analysis. If IV and EDVs or two collections with known parent-offspring relationships appear in the results at the same time, EDV and offspring were removed and replaced by a subsequent collection.

### 2.6. SNPs Markers for Rapidly Varieties Identification

In order to achieve rapid variety identification with fewer SNPs, based on 973 SNPs, according to the Perl method of Yang et al., 2019 [39] and Liu et al., 2019 [40], the discernibility of pairwise comparison of all samples was used as the first filter condition, and the dataset with the same discernibility was then selected with higher PIC. First, the highest discernible SNP loci were chosen as an initial dataset, and each SNP was subsequently added to the initial dataset to form a new dataset. The second SNP was chosen from the new datasets with the highest discernibility and was added to the initial dataset. The following selection was the same as the second SNP until the discernibility reached the maximum. Finally, a set of SNP markers with minimum numbers and high discernibility was selected for the rapid identification of varieties. Primers were designed in the two conserved flanking regions, and 10 samples were randomly selected for verification.

## 3. Result

### 3.1. Genome-Wide Perfect SNPs Discovery of 349 Tea Trees

A total of 829.05 G sequencing data were obtained from 349 tea trees, with an average of 2.38 G of data for each sample, with an average proportion of 98% Q20 and 93.05% Q30. A total of 5,737,108,966 sequences were obtained. There were 5,668,097,099 sequences that could be matched to the tea reference genome, with a matching rate of 98.83% (Table S1). These results indicated a high sequencing quality. After strict filtering, a total of 12,937,679 SNPs were obtained (Table S2). Raw data obtained by sequencing have been uploaded to the NCBI database (BioProject number: PRJNA924950).

### 3.2. Population Structure Analysis

**Figure 1:**
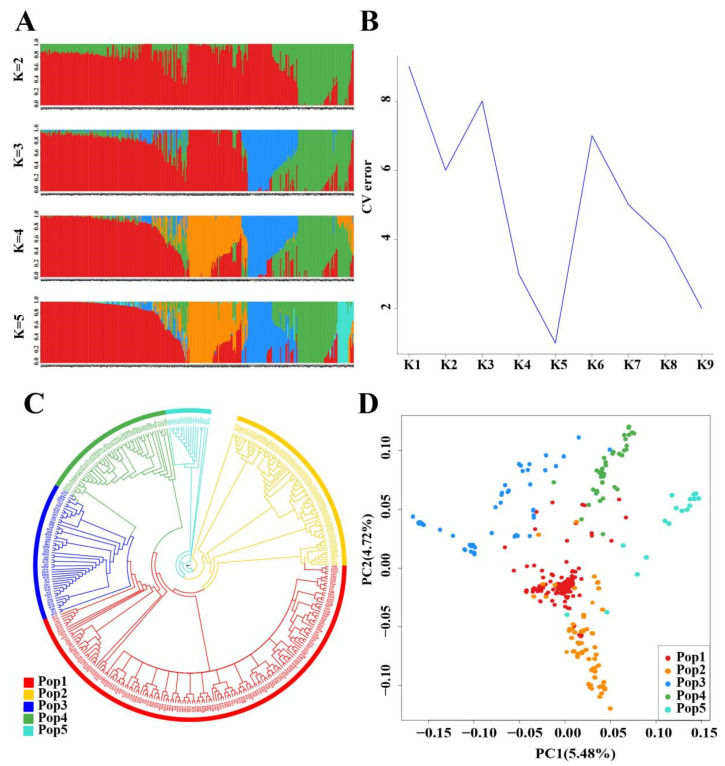
Population structure, unrooted neighbor-joining tree and principal component analysis of 349 tea trees based on the 12,937,679 perfect SNPs. (**A**) Population structure of the tea trees, with different K-values, based on Model-based clustering; (**B**) Cross-validation error (CV error); (**C**) Unrooted neighbor-joining tree of 349 samples; (**D**) Principal component analyses (PCA) of all populations.

The population structure of 349 tea trees was analyzed based on the 12,937,679 perfect SNPs. The model-based structure analysis showed that the best K value was K = 5 (Figure 1a,b). The phylogenetic analysis (Figure 1c) was in accordance with the population structure inferred by the structure analysis. In addition, PCA was conducted to assess the population structure (Figure 1d) and could indicate five clusters of tea trees, consistent with structure analysis at K = 5. All these suggested that the 349 tea trees could be classified into five populations. The presence of a mixture was observed within the five populations (the membership coefficient of its own population is less than 0.8). There were 159, 72, 51, 50 and 17 tea trees in populations 1–5, respectively (Table S3).

### 3.3. Core SNPs Set Exploration

**Figure 2:**
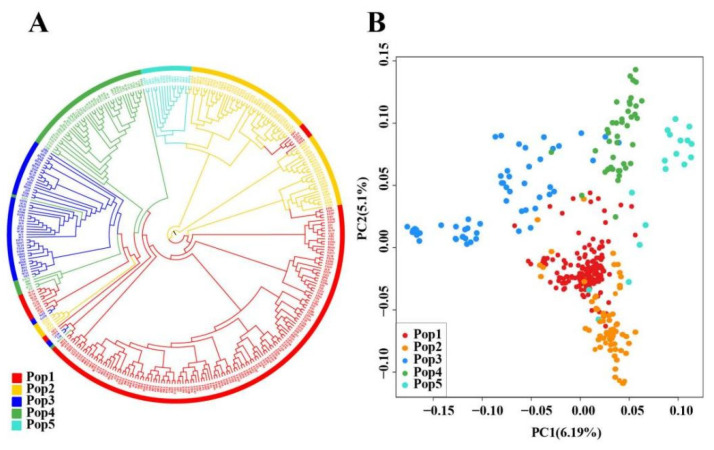
Unrooted neighbor-joining tree and PCA analysis based on core SNPs data set. (**A**) Unrooted neighbor-joining tree; (**B**) PCA analysis.

A set of 973 SNPs with MAF > 0.15, miss rate ≤ 5% and PIC > 0.15 was further selected as the core SNPs set (Table S4). Using these 973 SNPs, the phylogenetic tree analysis showed that the clustering relationships of the vast majority of tea trees were consistent with the clustering relationships using 12,937,679 SNPs, while only 21 individuals with extremely admixture backgrounds were inconsistent (Figure 2a). The result of the PCA analysis was also almost consistent with that using all 12,937,679 SNPs (Figure 2b). These results suggested that these 973 SNPs could almost represent the genetic diversity of 349 tea trees.

**Figure 3:**
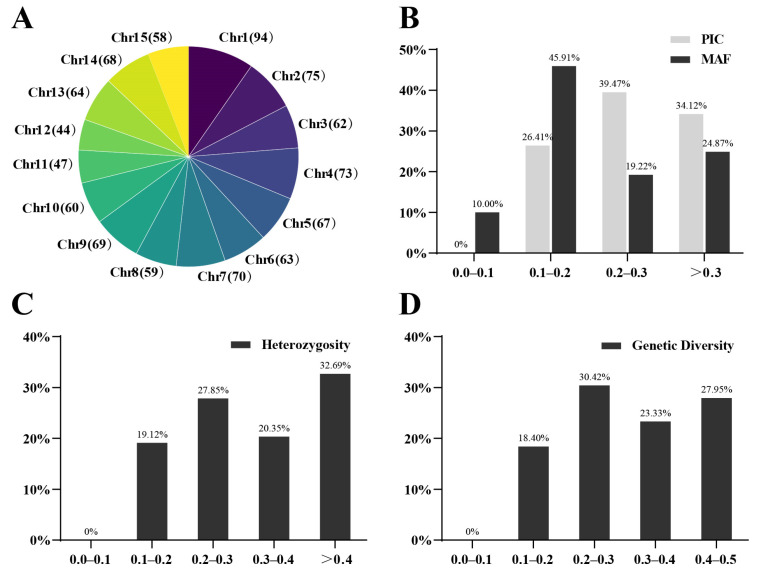
Genetic diversity analysis with 973 core SNPs in the 349 tea trees. (**A**)The number of 973 SNPs distributed on 15 chromosomes; (**B**) Polymorphic information content (PIC) and minor allele frequency (MAF); (**C**) observed heterozygosity (Ho); (**D**) genetic diversity (GD).

The number of the 973 SNPs distributed on chromosome 1 to chromosome 15 of the tea tree was 94, 75, 62, 73, 67, 63, 70, 59, 69, 60, 47, 44, 64, 68 and 58, respectively (Figure 3a). The PIC values of the 973 SNPs genotype in the 349 tea trees ranged from 0.150 to 0.375 with an average of 0.262, among which 34.12% SNPs had PIC values higher than 0.3. (Figure 3b). The average value of MAF was 0.223, among which the SNPs higher than 0.3 accounted for 24.87% (Figure 3b). The observed heterozygosity (Ho) displayed an average value of 0.355, with 53.04 % of all SNPs above 0.3 (Figure 3c). Moreover, the genetic diversity (GD) values of the 973 SNPs ranged from 0.163 to 0.5, with an average value of 0.319, among which 51.28% of SNPs were higher than 0.3 (Figure 3d).

### 3.4. GS and EDV Analysis

**Figure 4:**
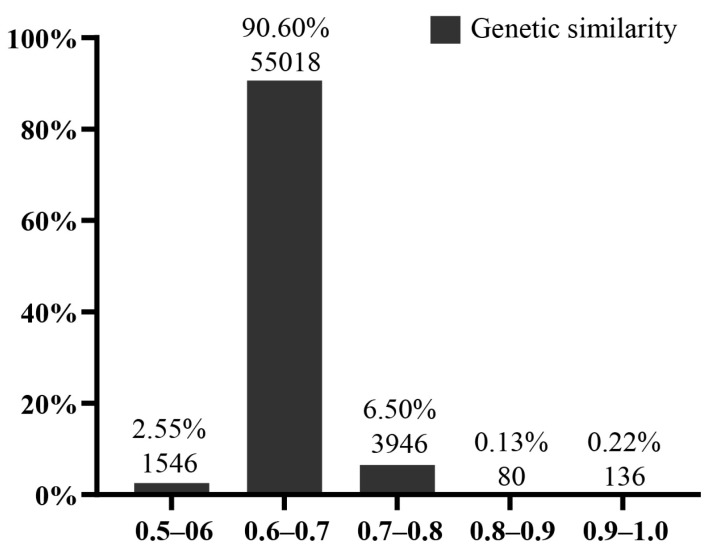
Genetic similarity statistics among 349 tea trees. The numbers in parentheses represent pairs of quantities.

A genetic similarity matrix calculating GS values between each sample was built within 349 tea trees (Table S5). Among 349 samples, 136 pairs (0.22%) had GS values higher than 0.9 (Figure 4). The GS values of six positive controls were set as reference thresholds for indisputable EDVs. In six positive controls, the GS value was 0.9764–0.9846. Therefore, a GS ≥ 0.97 was taken as a threshold for indisputable EDVs (Table S6). According to the time rated as varieties by the Tea Variety Certification Committee, the earlier varieties were set as IV, and the others were EDV. Among 349 samples, 121 have been registered as varieties (Including 63 national varieties and 58 provincial varieties), of which 22 are considered EDVs, and 19 are indisputable EDVs (Table 1). In addition, 38 landraces had essentially derived relationships with other tea trees, of which 30 were indisputable (Table S7).

### 3.5. Core Varieties Analysis

A genetic similarity matrix was built based on the number of differential SNP genotypes between each sample among 349 tea trees (Table S8). The top 20% of varieties with minimum differential SNP genotypes were core varieties within each population (Table 2). Finally, 70 tea trees were considered to be the core or backbone of 349 tea trees (Table S9).

### 3.6. Screening of SNPs Markers for Rapid Identification

**Figure 5:**
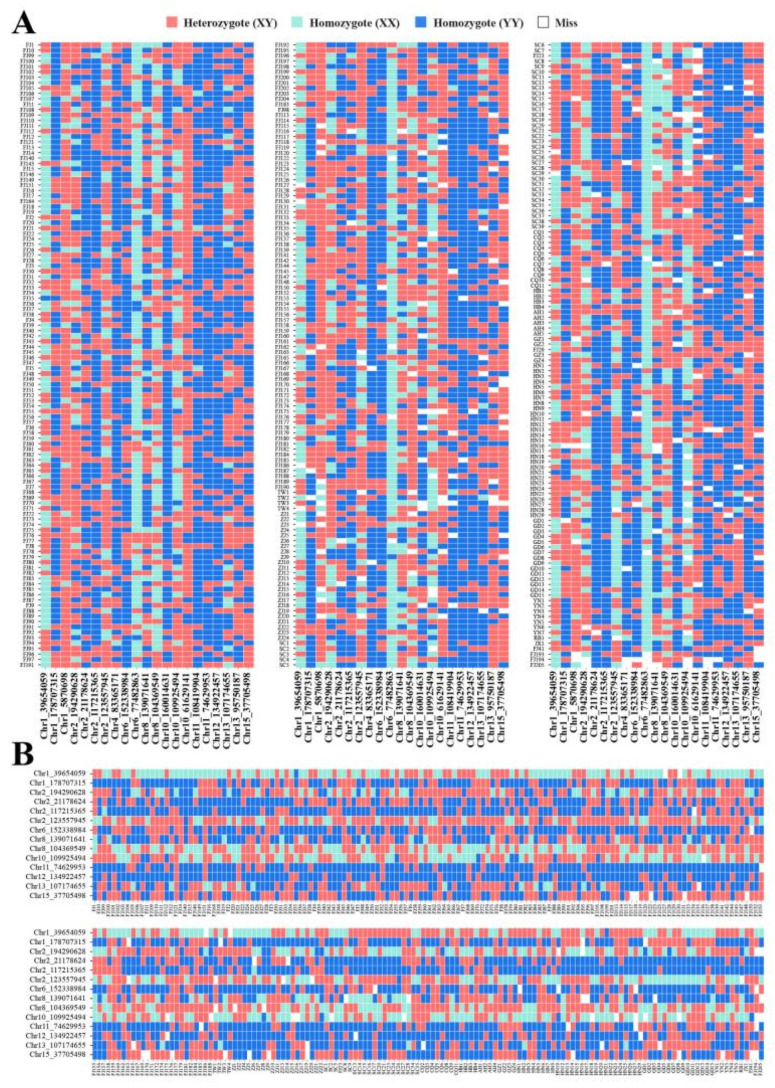
SNP Fingerprints of 349 tea trees. Each line represents one SNP locus, and each column represents one sample. Red, Green and Blue color represents the Homozygote (XY), Homozygote (XX) and Heterozygote (YY), respectively. Blank represent the Missing data. (**A**) Twenty-one SNPs perfectly differentiated 349 tea trees; (**B**) fourteen SNPs perfectly differentiated 279 non-EDV.

**Figure 6:**
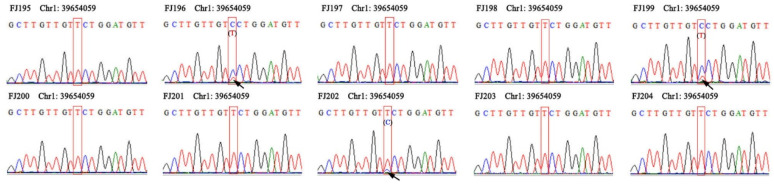
Primers were randomly verified by PCR. Randomly select 10 samples for first-generation sequencing and typing; arrows indicate clearly identifiable heterozygous sites.

By using pairwise comparison discernibility screening from 973 core SNPs, a set of 21 SNPs was selected for easier and faster study in 349 sample identification (Figure 5a), among which 14 SNPs could identify non-EDV (Table S10, Figure 5b). The PIC average value of 21 SNPs in 349 samples was 0.32, the Ho average value was 0.420, and the GD average value was 0.38 (Table 3). Twenty-one pairs of primers of SNPs markers were designed (Table 3 and Table S11) and randomly verified by PCR with good effect (Figure 6).

## 4. Discussion

In this study, a tea population of 349 tea trees from 12 provinces, including 63 National varieties, 58 Provincial varieties and 228 landraces, were collected for the first time to evaluate the essentially derived relationship between tea trees using GBS-SNPs. The GBS approach identified a total of 12,937,679 high-quality SNPs, and all samples passed the quality assessment with an average call rate of 98.8%. High numbers and high-quality SNPs suggested that the GBS approach is powerful for the genetic diversity analyses of tea species. Population structure and diversity analyses are important for the classification and identification of germplasm resources. Our population structure, PCA, and phylogenetic relationship analyses consistently showed that 349 tea trees could be divided into five populations. This was mainly based on the genetic relationship between tea trees but not on morphological characteristics of tree shape or leaf size, which was consistent with previous studies on the differentiation of wild and cultivated tea trees [41]. Thus, these five populations should be the groups representing different lineage sources. This also further suggested that distance coefficients measured using morphological data were of limited use in distinguishing between IVs and putative EDVs, that they did not always reflect lineages or genetic relationships, and that molecular markers have stronger discriminative power and are, therefore, more suitable for the identification of EDVs than morphology [42].

A suitable set of SNP markers for the implementation of the EDV system should be able to represent genetic diversity, cover the whole genome uniformly, and have a high discrimination capacity [42]. In this study, a set of SNP markers with an extremely low miss rate (≤5%) and PIC > 0.15 was selected that were evenly covered across the 15 chromosomes of the tea plant genome. A population genetic diversity analysis using 973 SNPs was almost consistent with all 12,937,679 SNPs, which suggested these 973 core SNPs were sufficient for representing the genetic diversity of the 349 samples. The relatively modest PIC average value and MAF average value might be due to the presence of EDVs in 349 tea cultivars, suggesting that breeding practices have an effect on reducing genetic diversity in cultivated germplasm [43]. A high Ho average value (0.355) and GD average value (0.3) suggested that these 973 perfect SNPs are informative with good discrimination capacity and suitable for the following variety identification.

What similarity ratio can be used as the threshold of EDV has been a controversial issue. CIOPORA (International Community of Breeders of Asexually Reproduced Horticultural Plants) recommended a genetic similarity coefficient of 0.9 as the standard [37,44]. However, a genetic similarity coefficient of 0.9 as the threshold standard may not be suitable for all crops, and a marker-based DUS (significance, uniformity, and stability) assessment system for EDV required a crop-by-crop approach [42]. At present, the specific threshold value of EDV for tea plants has not been clarified and needs to be further explored. In this study, we could only temporarily take the genetic similarity coefficient of 0.9 as the threshold value and regarded it as a reference for whether there was a certain derivative relationship between tea trees. In addition, even if individuals of the same variety have been cultivated in different places for a long time, there will be a small accumulation of genetic mutations. According to the examples given in the UPOV act, mutants were considered as EDVs [12,45]. If the genetic similarity between two individuals is extremely high, even greater than the genetic similarity between individuals of the same variety, but they have different phenotypes, they should be considered to be mutants with a derived relationship. Therefore, six pairs of tea trees known to belong to the same variety but grown in different places were used as positive controls to determine an indisputable derivation relationship, and the smallest genetic similarity value among them (0.97) was taken as the threshold for an indisputable EDV according to the “tail principle” [4,46]. If according to this criterion, 22 of the 121 registered varieties used in the study were EDVs (GS > 0.9), and 19 were indisputable EDVs (GS > 0.97). It was worth noting that some varieties with derived relationships come from different provinces, which means that a certain amount of EDVs might have been produced in the process of tea tree cross-breeding due to the extensive introduction of tea trees and excessive selection of elite varieties such as Fudingdahao, Tezao213, etc., and these results were also consistent with previous breeding records [41,47]. Further, in order to provide a reference for the selection of parents in the subsequent breeding process of tea trees, the core varieties in 349 tea trees were also analyzed by the least distance stepwise sampling method [38,39,40]. After excluding those varieties with possible derived relationships and known progeny varieties, the top 20% of the remaining varieties of each population were considered as the core/backbone varieties for the future breeding of tea trees.

In the application of SNP markers for tea varieties and EDVs, we conducted the first investigation of EDV in 349 tea trees using 973 SNPs screened by GBS technology, and the 349 tea tree samples used in this study were from a wide range of sources (12 different provinces and regions, including 63 national elite cultivars, 58 provincial elite cultivar and 228 landraces); therefore, this SNP set have certain application value in tea variety identification. In order to identify variety more conveniently and economically by using fewer SNP markers, a set of 21 SNPs was also furtherly screened from 973 SNP markers to identify 349 tea trees and DNA fingerprints were set up, of which only 14 SNPs could identify non-EDVs in 349 tea trees. Random validation of these 21 SNP markers showed good evaluation results, which could meet the requirements of efficient, flexible, simple, rapid and low-cost detection applications for these 349 tea trees in the future.

Furthermore, 973 SNP markers in this study have been strictly screened, and there is no other SNP interference on either side of these sites, so these SNP markers are very suitable for future detection of tea trees by SNaPshot technology, also known as mini-sequencing, a primer extension-based method developed for the analysis of SNPs [48]. Although the derivation threshold of the tea tree has not been definitively defined, the pairs of individuals with GS > 0.9 could be regarded as having a suspicious derivation relationship according to the recommendations of ISF [44]. In contrast, the pairs of individuals with a GS > 0.97 could be clearly regarded as a derivation relationship. Nevertheless, if this marker set is to perform future EDV identification in tea trees, further data needs to be collected in collaborative studies of genetic material obtained through different breeding methods or reference populations. In addition, it is also still necessary to combine pedigree relationship and morphological data so as to make a comprehensive EDV identification of tea trees.

## 5. Conclusions

In conclusion, this study was the first to investigate and analyze the EDV status of tea trees in China, and 60 varieties/strains were identified as EDVs, including 22 registered varieties (19 were indisputably EDVs). Based on the high discrimination capacity and genome coverage, our study provided a set of 973 SNP markers capable of identifying 349 tea trees, of which only 21 SNP markers were able to identify all 349 tea trees (14 SNP markers could be used for 100% identification of non-EDV). Our results provide a research basis for the genetic background analysis of tea trees in the process of molecular-assisted breeding and contribute to the implementation of the EDV system in the field of tea trees in the future.

## Figures and Tables

**Table 1:** Analysis of essentially derived varieties (GS > 0.9).

No.	IV	EDV	GS
	Sample ID, Name (Grade, Time)	Sample ID, Name (Grade, Time)	
1	FJ115, Fudingdahaocha (National, 1984)	FJ166, Fuyun595 (Provincial, 1988)	0.9744 *
2		FJ193, Jiulongdabaicha (Provincial, 1998)	0.9805 *
3		SC11, Shuke no.1 (Provincial, 2015)	0.9805 *
4		SC30, Gulinniupicha (Provincial, 1985)	0.9826 *
5		SC6, Mengshan no.9 (Provincial, 1989)	0.9815 *
6		SC7, Mengshan no.11 (Provincial, 1989)	0.9774 *
7	SC12, Tezao213 (Provincial, 2003)	SC10, Chuannonghuangyazao (Provincial, 2009)	0.9703 *
8		CQ1, Bayutezao (National, 2014)	0.9774 *
9		SC14, Chuanmu28 (Provincial, 2010)	0.9774 *
10		SC19, Huaqiu no.1 (National, 2014)	0.9754 *
11	AH5, Anhui no.3 (National, 1987)	SC39, Mengshan no.23 (Provincial, 1989)	0.9897 *
12		SC33, Mingshanbaihao131(Provincial, 1997)	0.9887 *
13	FJ178, Dayewulong (National, 1985)	FJ126, Jinmudan (National, 2010)	0.9344
14	FJ114, Fuyun no.6 (National, 1987)	SC25, Tianfuhong no.1 (Provincial, 2016)	0.9805 *
15	ZJ6, Biyun (National, 1987)	CQ7, Yucha no.2 (Provincial, 2001)	0.9190
16	CQ4, Shuyong no.2 (National, 1987)	CQ10, Shuyong703(National, 1994)	0.9374
17	CQ8, Shuyong no.3 (National, 1994)	SC3, Tianfucha no.28 (National, 2014)	0.9887 *
18	FJ113, Zimudan (National, 2010)	SC31, Ziye (Provincial, 2018)	0.9928 *
19	ZJ18, Zhenong902 (National, 2002)	ZJ15, Zhenong901 (National, 2020)	0.9815 *
20	HN11, Jianbohuang (Provincial, 1987)	HN5, Baihaozao (National, 1994)	0.9949 *
21	HN10, Yusun (Provincial, 1997)	HN8, Yulv (National, 2010)	0.9867 *
22	ZJ3, Yingshuang (National, 1987)	HN24, Jianghuakucha (Provincial, 1987)	0.9877 *

GS: the genetic similarity coefficient. Symbol * indicated that the GS value (GS > 0.97) implied an indisputable derived relationship.

**Table 2:** Analysis of core varieties in 5 populations.

Pop	Num (All)	Num (Core)	Sample Name (Sample ID)
Pop1	159	32	Qizhong-JPC (FJ205); Qidan (FJ192); 1105 (FJ198); Bantianyao (FJ50); Baisuixiang (FJ195); Beidou2 (FJ196); Jiulongqi (FJ201); Jinyaoshi2 (FJ199); Zuishuixian (FJ55); Huangjinya (ZJ5); Biyun (ZJ6); 0206 (FJ19); Dahongmei (FJ104); Bujiantian (FJ45); Laojunmei (FJ88); Zhengyulan (FJ59); Zhengliutiao (FJ100); Zhongcha108 (ZJ1); Yanzhiliu (FJ76); Xiannvsanhua (FJ39); 0207 (FJ20); Yucha no.2 (CQ7); Guilindan (FJ28); Zhenong12 (ZJ19); Liuxiangjianbuzhichun (FJ42); Shiru (FJ112); Xiaoyugui (FJ97); Tieluohan (FJ85); Baojinghuangjincha no.1 (HN6); Xiangtianmei (FJ67); Shanzhizi (FJ83); Luohanqian1 (FJ101)
Pop2	72	14	306 (FJ194); Zhengtaiyang2 (FJ202); Ziluolan2 (FJ203); Ruixiang (FJ122); Tieguanyin (FJ185); Chungui (FJ145); Zaoguanyin (FJ175); 0306C (FJ158); Dayewulong (FJ178); Baimaohou (FJ149); 0206-A (FJ134); Benshan (FJ184); Huangdan (FJ120); Rougui (FJ84)
Pop3	51	10	Fudingdahaocha (FJ115); Fudingdabaicha (FJ116); Mabianlv (SC5); Anhui no.3 (AH5); Mengshan no.23 (SC39); Baihaozao (HN5); yingshuang (ZJ3); Bixiangzao (HN7); Zhengbaihao (FJ31); Zhenong117 (ZJ20)
Pop4	50	10	Jianhexiangcha (SC18); Zijuan (YN7); Qianmei809 (GZ1); Dayelong (JX1); Qigaixian (FJ176); Xintianwandacha (HN15); Shuyong401 (CQ2); Shuyong no.3 (CQ8); Shuke no.36 (SC23); Bashanzao (SC26)
Pop5	17	4	Zhilanxiang (GD1); Chengmen (GD6); Laoxianweng (GD3); Wuyedangcong (GD14)
Total	349	70	

Pop: Population; Num: Number.

**Table 3:** 21 Primer information for 349 samples.

No	Name	SNP	Primer	Tm	Len	Site	PIC	Ho	GD
1	Chr1_5870698	G/T	F: AACACCGAGACGCTTTGGAT	58	385	279	0.44	0.83	0.54
			R: CCTCGCACCTCACACTTAGG						
2	Chr1_39654059	C/T	F: GTTGGAACCGATGCTAAT	52	426	78	0.32	0.43	0.37
			R: GCATTCAAAGCCAGAGTAA						
3	Chr1_178707315	C/T	F: TAGAAGTTCCGCAGCACG	52	369	157	0.28	0.36	0.32
			R: AGGTTCATGTACCACCAT						
4	Chr2_21178624	C/T	F: GCGTTTGTGCAAGGCTGTTA	58	382	312	0.27	0.36	0.31
			R: AGATGATGCCCTGCTAGCTC						
5	Chr2_117215365	C/T	F: ACGAAAGGCCGTTTCTGGAT	58	395	234	0.23	0.28	0.26
			R: ACTAGTTTGCCACCCCATCG						
6	Chr2_123557945	G/T	F: TCCCTCATCCCTCCTCAAGG	60	297	59	0.38	0.65	0.47
			R: CGACACAAATGGAGTCCGA						
7	Chr2_194290628	A/C	F: CCCATAGGACCGGACATCA	60	404	169	0.39	0.5	0.49
			R: AGCTTGGTTAGGGTCTTCGC						
8	Chr4_83365171	A/G	F: GGCTTAGTTAATGGTGAT	50	443	327	0.23	0.24	0.26
			R: GGAAGGTATGGGTTGTAT						
9	Chr6_77482863	A/G	F: TTTCCTCGTTTTGGTTAG	50	350	197	0.17	0.12	0.18
			R: ATATTTCGGCAAGGTTTA						
10	Chr6_152338984	C/T	F: AGCGTTGAAGCAGCATTTGG	60	510	286	0.26	0.31	0.29
			R: TTGTCCCAGTTGCAACAGGT						
11	Chr8_104369549	A/G	F: GCAAGCTCTATGTGCCTTGC	58	490	140	0.36	0.59	0.44
			R: GCCTTGTTGTGAAGCGAAG						
12	Chr8_139071641	C/T	F: AGCTCCGATATCCCTTGGGT	60	511	142	0.39	0.44	0.5
			R: GAGTTAAGGACCCTGTGCC						
13	Chr10_61629141	C/T	F: GTCGGGTCATCATCCGGATC	60	528	242	0.43	0.72	0.52
			R: GCAAGTGGCTTTCAGTCAGC						
14	Chr10_109925494	A/G	F: AGTGAGCTGGCACAAGTGTT	60	315	216	0.4	0.44	0.47
			R: AGCCCACTTTAGCACCATCC						
15	Chr10_160014631	C/G	F: TCCTGTTGAGTTGGGTAG	50	445	141	0.39	0.32	0.46
			R: ATCGTCCTTGGAATACTT						
16	Chr11_74629953	C/T	F: GCGAGCAATGTTTCCACGTT	60	396	339	0.21	0.23	0.23
			R: CGCACAAGCCTATTGCCTTG						
17	Chr11_108419904	C/T	F: CCAACTTGTTAGCCCCAAGG	60	464	192	0.27	0.24	0.29
			R: CTGTGGCAGGTCGACATCTT						
18	Chr12_134922457	A/G	F: AGCAGGAGCAGACACCTTT	58	513	216	0.19	0.22	0.21
			R: GAATTGGCACATGCTGCTCC						
19	Chr13_95750187	C/T	F: CTACCACCCCTAAGAGGCCT	58	452	80	0.4	0.63	0.49
			R: TTCTGCATCGCCTCGATACC						
20	Chr13_107174655	A/G	F: ATTTGAAGAATGCCGGGGC	58	323	140	0.35	0.38	0.42
			R: CCTCGCATCTCCTTTTCGGT						
21	Chr15_37705498	A/C	F: TGGGTATGGCTGCAAGATGG	60	537	131	0.41	0.55	0.48
			R: CCCAAACAAACACCCCCAT						
Average value							0.32	0.42	0.38

## Data Availability

Data will be made available on request.

## Supplementary Materials

The following supporting information can be downloaded at: https://www.mdpi.com/article/10.3390/plants12081643/s1. There are 11 supplementary tables, Table S1: 349 tea trees information and GBS resequencing data output statistics; Table S2: Quality filtering of GBS resequencing raw data; Table S3: Population structure analysis of 349 tea trees by using the model-based program STRUCTURE; Table S4: A set of 973 core SNPs for 349 tea trees; Table S5: A genetic similarity matrix by calculating GS values between each tea trees was built within 349 tea trees; Table S6: Accession pairs with essentially derived relationships (GS > 0.9) within 349 tea trees; Table S7: Analysis of essentially derived variety/strain within 349 tea trees; Table S8: A genetic similarity matrix was built based on the number of differential SNP genotypes between each sample among 349 tea trees; Table S9: Analysis of the core variety in 5 populations within 349 tea trees; Table S10: The 21 SNP markers polymorphism for 349 sample identification; Table S11: The 21 pairs of primers for 349 tea trees rapid identification.
